# Unconventional Extraction of Total Non-Polar Carotenoids from Pumpkin Pulp and Their Nanoencapsulation

**DOI:** 10.3390/molecules27238240

**Published:** 2022-11-25

**Authors:** Nicola Pinna, Federica Ianni, Francesca Blasi, Arianna Stefani, Michela Codini, Stefano Sabatini, Aurélie Schoubben, Lina Cossignani

**Affiliations:** 1 Section of Food Sciences and Nutrition, Department of Pharmaceutical Sciences, University of Perugia, 06126 Perugia, Italy; 2 Section of Pharmaceutical Chemistry and Technology, Department of Pharmaceutical Sciences, University of Perugia, 06123 Perugia, Italy; 3 Center for Perinatal and Reproductive Medicine, Santa Maria della Misericordia University Hospital, University of Perugia, 06132 Perugia, Italy

**Keywords:** pumpkin, carotenoids, extraction methods, HPLC-DAD analysis, solid lipid nanoparticles, in vitro antioxidant activity, functional foods, UHPLC-MS/MS, encapsulation, medicinal plants

## Abstract

Pumpkin is considered a functional food with beneficial effects on human health due to the presence of interesting bioactives. In this research, the impact of unconventional ultrasound-assisted extraction (UAE) and microwave-assisted extraction techniques on the recovery of total non-polar carotenoids from *Cucurbita moschata* pulp was investigated. A binary (hexane:isopropanol, 60:40 *v/v*) and a ternary (hexane:acetone:ethanol, 50:25:25 *v/v/v*) mixture were tested. The extracts were characterized for their antioxidant properties by in vitro assays, while the carotenoid profiling was determined by high-performance liquid chromatography coupled with a diode array detector. UAE with the binary mixture (30 min, 45 °C) was the most successful extracting technique, taking into consideration all analytical data and their correlations. In parallel, solid lipid nanoparticles (SLN) were optimized for the encapsulation of the extract, using β-carotene as a reference compound. SLN, loaded with up to 1% β-carotene, had dimensions (~350 nm) compatible with increased intestinal absorption. Additionally, the ABTS ((2,2′-azino-bis(3-ethylbenzothiazoline-6-sulfonic acid) assay showed that the technological process did not change the antioxidant capacity of β-carotene. These SLN will be used to load an even higher percentage of the extract without affecting their dimensions due to its liquid nature and higher miscibility with the lipid with respect to the solid β-carotene.

## 1. Introduction

Since ancient times, vegetables and fruits have always provided the possibility of a wide application field, ranging from textile manufacturing to medicinal applications, in addition to food use. Phytochemicals present in plant foods are key components contributing to ameliorating daily dietary habits being interesting sources of functional compounds for humans [1]. Most of their beneficial health properties are ascribable to bioactives, commonly produced by the secondary metabolism, which shows various effects, including antioxidant, anti-obesity, and anti-hypertensive [2]. As a consequence, in the nutraceutical and pharmaceutical fields, there is an increasing interest in searching for and identifying new bioactive molecules and applying innovative extraction methods to isolate them from plant food and agri-food waste. The increment in urbanization and plant-based food utilization has led to a rapid accumulation of biomass residues, requiring the development of sustainable procedures for their valorization [3]. 

The recovery of these bioactives for potential use in food, pharmaceutical, and cosmetic industries has aroused an increasing interest from researchers. To this aim, different extraction methods can be applied [4]. Generally, the isolation of carotenoids from plant-based foods can be carried out by numerous techniques based on solid–liquid extraction, split into conventional and unconventional ones [4]. Among traditional ones, maceration usually uses mild heating treatment to improve the efficiency of extraction, besides being time- and solvent-consuming. These methods are easily usable, but a high-solvent consumption is the main weakness. On the contrary, the main strength of unconventional extraction techniques (i.e., ultrasounds, microwaves, and supercritical fluids, to cite a few) is the possibility to improve extraction efficiency and/or selectivity by using processing aids/energy inputs [5].

Pumpkins (*Cucurbita* spp.) are vegetables belonging to the Cucurbitaceae family, with both wild and domesticated species. The most common are *Cucurbita maxima*, *C. pepo*, and *C. moschata*. They are native to Central and Southern America but are grown and widely consumed all around the world [6]. Pumpkins are a rich source of carotenoids, among which β-carotene, a vitamin A-precursor with antioxidant properties. It is present mainly in pulp, filaments, and peel, along with other bioactive compounds, such as phenol compounds [6]. A plethora of epidemiological studies have reported that high dietary carotenoid intake is related to a lower risk of chronic diseases such as cardiovascular, neurological, and eye-related diseases [7]. Though carotenoids are fat-soluble compounds with high instability to light and oxygen, they have very poor bioavailability from natural sources and considerably limited absorption in vivo [8,9]. Their absorption by enterocytes occurs mainly via passive diffusion, and their emulsification and micellization are key for their absorption by intestinal cells. Therefore, all these factors limit the application of carotenoids as ingredients in food systems [10,11,12]. To improve the bioaccessibility and bioavailability of carotenoids, some strategies must be applied to integrate carotenoids into foods and beverages. For instance, their encapsulation within micro- or nanostructures can be a solution to overcome these hurdles. Thanks to the small dimensions and the high surface area, nanocarriers are able to: (i) increase the solubility and, consequently, the bioavailability of carotenoids; (ii) protect them along the gastrointestinal tract; (iii) control their release during digestion and their delivery to specific tissues; (iv) make them more stable during manufacturing processes and storage. In this context, nanotechnology can be used to mask unwanted flavors and to ameliorate the homogeneity, texture, and mechanical and thermal properties of the food system [13]. Solid lipid nanoparticles (SLN) are ideal for the food field since they are composed of food-grade ingredients, as opposed to many polymers, emulsifiers, and organic solvents commonly used in the production of nanocarriers. They offer great versatility in physicochemical properties (e.g., size, load, crystalline habit) according to the selected lipid. Moreover, the use of nanosized particles allows better compatibility with the food matrix avoiding phase separation [14]. 

In the present study, innovative extraction techniques such as ultrasound- and microwave-assisted extraction (UAE and MAE, respectively) methods were employed to obtain total non-polar carotenoid-rich extracts from pumpkin (*C. moschata*) pulp. A comparison with a traditional technique, maceration (MAC), was also performed. Binary (hexane:isopropanol, 60:40 *v/v*) and ternary (hexane:acetone:ethanol, 50:25:25 *v/v/v*) mixtures were tested. All extracts were characterized for their in vitro antioxidant properties and carotenoid composition. The profiling of isolated compounds was studied by reversed phase-high performance liquid chromatography coupled with a diode array detector (RP-HPLC-DAD). SLN were selected as nanocarriers to encapsulate carotenoids, and in particular, β-carotene was used as a reference compound. The findings are expected to have applications in various industrial sectors such as food, pharmaceutical, and cosmetic.

## 2. Results and Discussion

### 2.1. Characterization of C. moschata Pulp Extracts

Plant-foods typically contain both non-polar carotenes and more polar xanthophylls. An optimal extraction procedure should bring these bioactives into solution without modifying their chemical characteristics and biological properties. For this reason, extraction should immediately follow the cutting of the vegetable tissue, as this leads to the release of enzymes (e.g., lipoxygenase), which catalyze both carotenoid oxidation and double bond isomerization [15]. For this reason, in this research, immediately after the cutting, the little pulp pieces were dried in mild conditions (40 °C) and then immediately minced to obtain a powder. Only after this careful sample preparation, the UAE, MAE, and MAC procedures were carried out following the extraction conditions reported in Section 3.3. 

It has been reported that dried materials can be extracted with water-immiscible solvents, but extraction is usually more efficient if the samples are first rehydrated and then extracted with water-miscible solvents [15]. In the case of soft fruits/vegetables, the use of a fast mechanical blender efficiently disrupts and homogenizes plant cells. In the case of harder or gummy samples, instead, a previous soaking in the extraction solvent to soften the cell wall may also be required. Prolonged soaking must be avoided to prevent carotenoid isomerization and degradation [15]. Therefore, in this research, before each extraction, the pumpkin pulp powder was subjected to a static soaking, followed by a gentle vortex action, in order to guarantee the solvent penetrated the vegetable matrix and softened the cell wall.

At this point, total non-polar carotenoids of pumpkin pulp were extracted by two unconventional techniques, UAE and MAE. These were selected because there is currently an increasing demand for extraction techniques with improved yield, a shorter time, and reduced organic solvent consumption. Organic solvents (e.g., hexane, acetone, ethanol, benzene, chloroform, and petroleum ether) are commonly used in carotenoid extraction, but a mixture of solvents (hexane:isopropanol; hexane:acetone:ethanol), at a different ratio, is often employed. A comparison with a traditional method (MAC) was also carried out. In this regard, in order to evaluate the impact of the solvent on the recovery of the carotenoids from pumpkin pulp powder, a binary mixture (hexane:isopropanol, 60:40 *v/v*) and a ternary mixture (hexane:acetone:ethanol, 50:25:25 *v/v/v*), consisting of fully miscible solvents, were comparatively tested. The times of extraction were chosen on the basis of our previous results [16]. The type of solvent on the basis of other research papers regarding the extraction of carotenoids from pumpkin peel and pulp [17] and from tomato waste [18,19]. Sharma and Bhat [17] used the same binary mixture (hexane:isopropanol, 60:40 *v/v*) to extract carotenoids from two varieties of pumpkin (Var. Gold Nugget and Var. Amoro F1). The presence of acetone in the ternary solvent mixture did not significantly affect the carotenoid extraction in a positive way. This conclusion was also reported by Luengo et al. [18]. Sometimes this ternary mixture was used for the extraction of *all-trans* lycopene by enzyme-assisted extraction [19]. 

Before deepening into the chemical and biological aspects of extracts, it must be pointed out that the mechanism of action of the two unconventional extraction techniques (UAE and MAE) is completely different. UAE mechanism is based on cavitation that enhances cell wall destruction, promoting the release of the bioactive compounds into the solvent. The MAE process, instead, is based on electromagnetic radiations that induce heating via dipolar rotation and ionic conduction of the molecules, thereby weakening or breaking cell walls.

Table 1 shows the results of the characterization of the different extracts: the extraction yield (%), the total carotenoid content (TCC), and the in vitro antioxidant activities, determined by ABTS (2,2′-azino-bis(3-ethylbenzothiazoline-6-sulfonic acid) diammonium salt), and ORAC (Oxygen Radical Absorbance Capacity) assays. 

The percentage of extraction (yield, %) was calculated using the following equation:

Yield, % (g/100 g) = (W_1_ × 100)/W_2_ (1)

where W_1_ is the weight of the extract residue obtained after solvent removal, and W_2_ is the initial weight of pumpkin powder.

As regards yield (%), it is possible to observe that the binary solvent gave similar values of yield (not statistically different, *p* ≥ 0.05) using all three types of extraction techniques (UAE, MAE, and MAC). Similar results were obtained by Chuyen et al. (2018) [20]; in fact, the authors observed that MAE and UAE did not show any significant improvement in carotenoid extraction yield with respect to MAC. Since the yield results were determined by the gravimetric method, this parameter may not be a sufficient reason to choose this extraction method. For this reason, other analytical characterizations (spectrophotometric and chromatographic ones) were also carried out. 

At first, the TCC was determined by spectrophotometric assay setting the instrument at 450 nm, using the standard calibration curve of β-carotene. Using both unconventional techniques (UAE and MAE), the binary mixture gave a higher content of TCC (145.33 and 126.02 μg/g for UAE and MAE, respectively) with respect to the ternary mixture, while using the conventional MAC the best result (162.81 μg/g) was not obtained with the binary, but with the ternary mixture. Regarding TCC values, only statistically significant results (*p* ≤ 0.05) were obtained from a comparison between the TCC of extracts obtained with ternary solvent by using MAE and MAC. 

On the basis of the results of TCC, MAE would not be the method of choice for the extraction of total non-polar carotenoids from pumpkin pulp, while UAE is advantageous with respect to MAC when a binary mixture is chosen. However, other analytical parameters will be taken into consideration to make a deeper and definitive evaluation of all extraction techniques.

Sharma and Bhat [17] found values of TCC from 26.98 to 32.69 μg/g of oil extract, so the values are not comparable, but they confirmed the trend that UAE gave the highest TCC values with respect to MAE and conventional extracts. On the basis of their results, the apparent extraction trend observed for all the analyses (antioxidant and coloring) from different extraction techniques was UAE > MAE > MAC. 

It is known that some carotenoids show pro-vitamin A activity, but their capacity to act as singlet oxygen quencher and scavenger peroxyl radicals by conjugated double bonds also provide them powerful antioxidant properties [21]. Therefore, in vitro spectrophotometric assays were carried out to evaluate this capacity. 

No ferric-reducing activity power (FRAP) was observed for all extracts. This result was in line with previous studies [22,23] where the authors found that none of four β-carotene isomers (all-trans, 9 cis, 13 cis, and 15 cis) showed ferric-reducing activity under the used conditions, which supports the previous findings of Pulido and co-workers [23]. This may be due to the circumstance that the ferric ion is incorporated into the steric-demanding di-tripyridyl triazine complex. However, the reactive part of carotenes (both in α and β) is the conjugated polyene chain in the center of the molecule, making it difficult for steric-demanding oxidants to interact with the carotenoid, especially with the bicyclic structures. 

Due to this result of the FRAP assay, the ability of β-carotene to undergo single electron transfer-based reactions was utilized in the analysis of ABTS^●+^ bleaching activity. The ABTS assay was carried out on all extracts and it can be seen that the ABTS value of UAE extract obtained with the binary mixture was significantly (*p* ≤ 0.05) higher than MAE extract (958.88 μg TE/g vs. 505.01 μg TE/g, respectively). Using the ternary mixture, the ABTS value was the highest for MAC extracts (3697.62 μg TE/g), followed by UAE, and MAE (2332.32 and 1870.03 μg TE/g, respectively), showing statistically different results (*p* ≤ 0.05). The results of the ABTS assay support the view that MAE would not be the unconventional method of choice for the extraction of total non-polar carotenoids from pumpkin pulp, both using binary and ternary mixture. Using the DPPH assay, similar results to the FRAP assay were obtained. This is one of the most popular assays for the evaluation of the antioxidant capacity of compounds due to its simplicity, low cost, and relative speed, yet some drawbacks are possible. Among these, it is important to remember that: the DPPH-antioxidant reaction is reversible to some extent; therefore, the measured antioxidant capacity may be lower than it should be; DPPH reagent shows low accessibility due to high steric hindrance; therefore, large molecules may not be able to access the DPPH radical site [24]. Some authors reported that the ABTS assay is more responsive than DPPH for the evaluation of the antioxidant activity of carotenoid extracts because ABTS correlates positively with their free radical scavenging activity compared to the DPPH assay [25]. All these considerations additionally support our DPPH results.

Finally, the total antioxidant capacity (TAC) was assessed using an ORAC assay. The ORAC value was the highest for UAE extract (2832.76 μg TE/g) obtained using binary mixture, while the data of ternary confirmed the trend of ABTS results. No statistically different (*p* ≥ 0.05) ORAC results were obtained when a comparison was made within the same extraction technique using different solvent mixtures. Statistically different results (*p* ≤ 0.05) were obtained from the comparison between ORAC values of MAE with UAE and MAC values.

ABTS and ORAC results, together with TCC data, show that the extract is potentially rich in bioactives (i.e., carotenoids); therefore, an HPLC-DAD procedure was also performed in order to characterize and quantify the carotenoids of extracts. A UHPLC-MS/MS technique was also carried out for the structural confirmation of the analytes. 

### 2.2. RP-HPLC-DAD Analysis

The extraction efficiency provided by the three techniques was monitored by RP-HPLC-DAD. In this frame, the first attention was paid to the set-up of an efficient chromatographic separation method able to selectively analyze the main species constituting the pumpkin pulp extract. In particular, the aim of this part of the study was to develop a methodology that would allow fast and effective separation and quantification of the investigated total non-polar carotenoids, as well as their separation from matrix components. 

β-carotene, lutein, and zeaxanthin standards were selected for the purpose, being among the main and most recurrent carotenoids in different varieties of pumpkin pulp [26,27]. 

**Figure 1:**
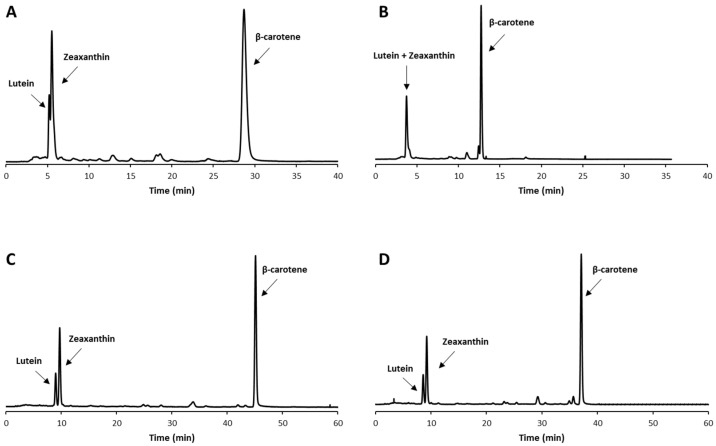
Chromatographic profiles of lutein, zeaxanthin and β-carotene standards with different gradient elution programs: (**A**) 0 min: 100% methanol, 0–30 min: 65% methanol and 35% MTBE, 30–60 min: 30% methanol and 70% MTBE, 0’: 100%A, 11, flow rate 1.2 mL/min; (**B**) 0 min: 80% methanol/water (97/3) and 20% MTBE, 0–2 min: 60% methanol/water (97/3) and 40% MTBE, 2–14 min: 60% methanol/water (97/3) and 40% MTBE, 14–27 min: 100% MTBE, flow rate 1.0 mL/min; (**C**) 0 min: 100% methanol/MTBE/water (82/16/2), 0–45 min: 60% methanol/MTBE/water (82/16/2) and 40% methanol/MTBE/water (25:75:2), 45–55 min: 100% methanol/MTBE/water (25:75:2), 55–60 min: 100% methanol/MTBE/water (25:75:2), flow rate 1.0 mL/min; (**D**) optimized experimental conditions reported in Section 3.6.

Starting from the experimental conditions applied in a previous study [28] and with the aim of improving the system chemoselectivity (Figure 1A,B), a preliminary optimization of the mobile phase composition was conducted. One main fault often reported in the literature is the close migration of structural isomers such as zeaxanthin and lutein under several chromatographic conditions, particularly when C18 columns are employed [29,30]. An improvement in this sense could be achieved through the use of a C30 column which has been found to be more effective for separating carotenoid isomers [31]. 

The fine-tuning stage of the experimental conditions in the RP-HPLC method development fulfilled our purpose. More in detail, several gradient programs, characterized by subtle variations of both organic components, were scrutinized and gradually modified until reaching the baseline separation between the investigated species in usable retention times. Figure 1C,D shows the chromatogram with satisfactory resolution obtained under the final gradient conditions set. The variation of the gradient, according to an OVAT (one-variable-at-time) approach, also resulted in beneficial smoothing of the jagged profile and the baseline noise encountered in the first chromatographic attempts.

**Figure 2:**
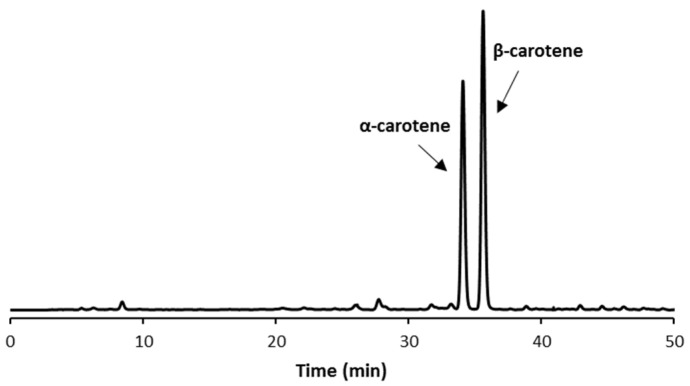
Chromatogram of the HPLC-DAD analysis of pumpkin pulp UAE extract.

Once established the optimal experimental conditions as shown in Figure 1D, the qualitative and quantitative profile of the pumpkin extracts was then evaluated. The HPLC-DAD profiles clearly evidenced the presence of two main peaks (Figure 2). The first peak was characterized by a retention time of around 35.5 min: according to data available in the literature, its identity was plausibly attributed to the α-carotene isomer [29,32] and successively confirmed and characterized by LC-HRMS analysis (see Section 3.7 for details). The second detected peak was established as β-carotene based on the correspondence between its retention time (approximately 37 min) and that of the reference standard. 

Small-scale isolation of both peaks was carried out through successive injections of the extract until obtaining 140 µg of α-carotene and 190 µg of β-carotene. The structural determination of the isolated species was checked via HRMS investigations. The fragmentation profiling by MS/MS confirmed the expected product ions at *m*/*z* 444 and *m*/*z* 119 related to the fragmentation of β-carotene as recently reported by Okada et al. [33] (see Supplementary Material for details). More in detail, the fragment ion spectrum for the β-carotene standard and for the β-carotene isolated by HPLC are shown in Figures S1 and S2, respectively. The fragmentation pattern of the α-carotene isomer was characterized by the presence of the diagnostic ion with *m*/*z* 388 [34] (Figure S3).

Very few traces of lutein were found only when a high extract concentration (>1.0 mg/mL) was injected, resulting in detector saturation and yielding a broad flat top peak of β-carotene. In line with data reported in the literature, Provesi et al. [35] reported a trend comparable to our findings. The carotenoid composition in raw *C. moschata* varieties presented a higher content of α-carotene (12.60 ± 1.56 µg/g) and β-carotene (19.45 ± 2.55 µg/g) compared to lutein content. Similarly, Azevedo-Meleiro and Rodriguez-Amaya [36] reported an increase in α- and β-carotene levels and a decrease in lutein in *C. moschata* varieties, parallel to the ripening stage of the vegetable.

### 2.3. Quantitation of Carotene in the Investigated Extracts

The quantitation of both carotenoids identified in all the investigated extracts was carried out on the basis of a calibration curve built up using standard solutions of β-carotene with concentration values in the range specified in Table 2.

The regression model provides good linearity (R^2^ = 0.999), revealing useful for predictive purposes. The established RP-HPLC method was further validated at a research level in terms of accuracy, precision, limit of detection (LOD), and limit of quantification (LOQ) (Table 2 and Table 3). This internal validation, generally pursued to assess new methods developed in-house at a basic level, refers to the concept of “fitness-for-purpose” and showed the method suitability to be applied to the present case.

High recovery % values (from 102.34% up to 105.68%) and a low range of variation of the RSD% values (from 0.60% up to 3.42%) were observed when the short-term (intra-day) accuracy and precision were evaluated, respectively. Appreciable recovery % (104.46%) and RSD% (0.11%) values were also computed in the long-term (inter-day) period. The outcomes achieved with the validation process, together with the obtained low LOD (1.07 ng/mL) and LOQ (3.24 ng/mL) values, revealed the adequacy of the analytical method to be applied for quantitative purposes. In accordance, this allowed the reliable quantitation of the two main forms of carotene, the extraction of which was the focus of the present study (Table 4). 

A correlation study was also carried out, considering all the extracts and all analytical parameters. An excellent correlation was obtained among all analytical results for extract obtained with the binary mixture, for example, between HPLC data (sum of α- and β- carotene) vs. TCC, ABTS, and ORAC values, as well as between ABTS vs. ORAC, and TCC values, and between TCC vs. ORAC. On the contrary, only some good correlations were obtained for the ternary mixture (HPLC data vs. TCC, and ORAC; ABTS vs. TCC); in fact, some unsatisfactory correlation values were observed (ORAC vs. TCC; HPLC data vs. ABTS), in particular when the correlation was evaluated between ABTS and ORAC.

Taking into consideration the results of spectrophotometric and chromatographic characterization of extracts together with the correlation data (Table 1, Table 4 and Table 5), UAE coupled with the binary mixture can be considered a promising strategy to extract total non-polar carotenoids (α- and β-carotene) from pumpkin pulp. Moreover, the choice of UAE was also supported by the consideration that UAE showed lower environmental impact effects than conventional MAC and MAE [37]. Finally, the presence of acetone in the ternary solvent mixture did not significantly affect in a positive way the extraction of carotenoids by unconventional methods, as also confirmed by other authors [17,18].

### 2.4. β-Carotene-Loaded Solid Lipid Nanoparticles Characterization

The most obvious requirement for a delivery system is its safety. SLN meet this need because they are composed of lipids, which are essential nutrients of the human diet and show good tolerability [38]. Lipid-based carriers are able to increase the bioaccessibility and the bioavailability of carotenoids [25]. In fact, nanoparticles can enhance carotenoid absorption by improving their solubility compared to the raw crystalline compound thanks to the greater surface-to-volume ratio, by prolonging the retention time, or through the direct absorption of the delivery systems. SLN also show better loading stability during storage and digestion with respect to other nanocarriers [39]. The solid-state of SLN is another important feature that guarantees the stability of the load limiting its mobility and exposure to oxidant agents [40,41].

The SLN prepared in this work were composed of hydrogenated sunflower oil (HSO), characterized by a melting temperature ~75 °C. β-carotene bioaccessibility will be influenced by the extent of lipolysis of the constituents of SLN and on the selected lipid. For instance, SLN composed of MCT (medium chain triglycerides) and hydrogenated palm oil (HPO) at different ratios present a greater bioaccessibility compared to that of nanoparticles made of MCT and glyceryl stearate. The former also provided an increasing β-carotene concentration in the micelle fraction during in vitro studies with the increase in HPO content [42]. To avoid the use of HPO, HSO, characterized by low values (reported on bulletin of analysis) of free fatty acids (0.07% as oleic acid), index of peroxides (0.5 mEq O_2_/kg), and index of iodine (2.3 g I2/100 g), and therefore by good oxidative stability, was used. Being made of 100% HSO, β-carotene accessibility should be high. In fact, sunflower oil is mainly composed of unsaturated long-chain fatty acids (i.e., linoleic and oleic acids) that can easily form micelles with high solubility, thereby increasing bioaccessibility [43,44]. Additionally, particular attention was paid to the choice of the emulsifier and co-emulsifier, which were soy lecithin and sodium cholate, respectively. In fact, they play an important role in the uptake of β-carotene by enterocytes once in the gastrointestinal tract. Cholic acid is a primary unconjugated bile acid and is an endogenous surfactant. Bile salts have the main role of solubilizing dietary lipids and liposoluble vitamins. Soy lecithin is an emulsifier that stabilizes SLN and forms vesicles and mixed micelles with sodium cholate. 

SLN were analyzed using dynamic light scattering (DLS), a widely used technique to determine particle size and size distribution of submicron particles. To obtain suitable SLN dimensions, the ideal lipid concentration was 2% *w*/*v,* and the optimized combination of the emulsifier and the co-emulsifier consisted of 0.8% *w*/*v* soy lecithin and 0.3% *w*/*v* sodium cholate. The blank SLN obtained in these conditions were characterized by two populations of nanoparticles (INTENSITY-WT): 2.6% of the sample had a mean diameter of 40 nm (38.9 ± 5.0 nm) and 97.4% had a mean diameter of 240 nm (239.1 ± 44.5 nm).

The particle population with smaller dimensions of about 40 nm was hypothesized to be micelles of sodium cholate, mixed micelles of sodium cholate, and lecithin or lecithin vesicles. It is essential to encapsulate β-carotene within the lipid nanoparticles, preventing it from being embedded within micelles or vesicles. Hence, the formation of different supramolecular organizations solubilizing β-carotene needs to be limited since they do not behave as SLN (e.g., release kinetics, loading stability). The emulsifiers should only stabilize the SLN. For this reason, the concentration of sodium cholate was reduced as much as possible with the aim of minimizing the presence of micelles and mixed micelles while ensuring stability. Additionally, soy lecithin was added to the molten lipid rather than to the aqueous phase to favor its incorporation in the lipid matrix. This is possible thanks to lecithin’s low HLB value and chemical structure [45].

β-carotene affected the original dimensions of SLN that were progressively larger, encapsulating increasing amounts of the carotenoid (Table 6).

While a minor load had a smaller impact on particle diameter, SLN achieved the largest size by loading up to 10% *w*/*w* β-carotene. In the suspensions of SLN containing 5% and 10% β-carotene, red aggregates were observed, probably ascribed to non-encapsulated β-carotene. These clusters were larger for 10% loaded SLN. Moreover, the aqueous dispersions were orange, assuming darker shades at higher β-carotene content due to the coloring power of the carotenoid.

Since some non-encapsulated β-carotene was readily observed in 5 and 10% loaded SLN, successive characterization was conducted only on 0.5 and 1% β-carotene loaded nanocarriers. In particular, Table 7 shows the experimentally determined content and corresponding β-carotene encapsulation efficiency of SLN determined on three different batches prepared on different days.

As shown by the data reported in Table 7, nearly 90% of β-carotene added to the lipid phase ended up in the 0.5% *w*/*w* loaded SLN suspension. On the other hand, a lower encapsulation efficiency of about 75% was registered when 1% *w*/*w* of β-carotene was added to the lipid matrix, probably because some β-carotene was lost during the production steps.

In order to assess whether the technological process and in particular HPH, had a negative impact on the antioxidant capacity of β-carotene encapsulated in the SLN, ABTS spectrophotometric assay was performed. SLN suspensions containing β-carotene were analyzed, and data were compared to those of the sole β-carotene and of soy lecithin. In this study, both the radical cation ABTS^•+^ powder and the antioxidant were incorporated in the same lipophilic phase to optimize the assay, in agreement with the methodology reported by Durmaz [46]. This was possible because the radical ABTS^•+^ is soluble both in water and organic solvent. However, it is necessary to first generate the reactive species in water and then lyophilize the solution because of the poor solubility of potassium persulfate and non-radical ABTS in organic solvents. The advantage of working with an organic solution is that the full antioxidant activity of the encapsulated substance can be assessed. In fact, working with the SLN aqueous suspension, only the substance on the surface or released from SLN can be assayed. For instance, Campos et al. observed that the antioxidant activity of loaded SLN was lower than that of free phenolic compounds. This was because SLN create a shell around the phenolic compounds protecting them against degradation and oxidation but also avoiding their interaction with the ABTS^•+^ radical cation [47].

The antioxidant capacity evaluated with the ABTS assays is reported in Table 8. The antioxidant capacity of the samples was expressed as mg of Trolox equivalents/mg SLN and determined from a calibration curve prepared with Trolox solutions previously treated by applying the same procedure as for the real sample. A calibration curve was prepared, and the correlation coefficient was 0.9985.

The relatively low RSD% obtained from three different batches of SLN demonstrated that the production process was reproducible. Furthermore, it was interestingly noted that a batch tested 2 months after its production gave results comparable with those derived from freshly prepared SLN. Thus, the formulation was allowed to preserve the antioxidant power over time.

Concerning soy lecithin solution, a negligible reduction in the absorbance of ABTS^•+^ and, consequently, a low value (0.008 ± 0.002) of mg of Trolox equivalents/mg lecithin was registered. Hence, the antioxidant activity was ascribable to β-carotene that showed 7.385 ± 0.021 mg of Trolox equivalents/mg β-carotene.

**Figure 3:**
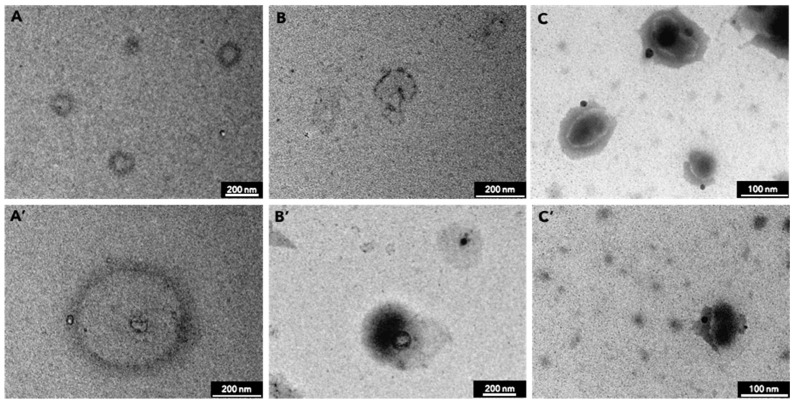
TEM photomicrographs of blank SLN (**A**,**A’**) and loaded SLN with 0.5% (*w*/*w*, **B**,**B’**) and 1% (*w*/*w*, **C**,**C’**) β-carotene, respectively.

TEM photomicrographs of SLN prepared with and without β-carotene are shown in Figure 3. SLN appear spherical with a smooth surface. The TEM photomicrographs show particle mean diameters compatible with those determined by DLS. Small differences are ascribed to the fact that photon correlation spectroscopy measures the mean hydrodynamic diameter, while from the TEM images, the diameter of dry particles was determined.

## 3. Materials and Methods

### 3.1. Plant Materials

Pumpkins (*C. moschata*) were collected in October 2021 in Umbria (central Italy). The pulp of pumpkins was separated manually and chopped into small pieces. Then, they were dried in a ventilated oven (Binder, Series ED, Tuttlingen, Germany) at 40 °C until a constant weight was reached. Finally, dried pieces were grounded in a blender and passed through a 250 µm sieve to obtain a fine powder (moisture 10 ± 1%). These samples were stored in amber glass containers away from light and humidity at room temperature until extraction. 

### 3.2. Reagents

2,2′-azino-bis(3-ethylbenzothiazoline-6-sulphonic acid) diammonium salt (ABTS), (±)-6-hydroxy-2,5,7,8-tetramethylchromane-2-carboxylic acid (Trolox), 2-methylpropionamidine)dihydrochloride (AAPH), and fluorescein sodium salt were from Sigma-Aldrich (Milan, Italy). Ultrapure water, methanol, acetonitrile, isopropanol, formic acid, and methyl tert-butyl ether (MTBE) of HPLC and UHPLC-MS/MS grade were purchased from Carlo Erba Reagents (Milan, Italy). The other solvents (hexane, isopropanol, ethanol, acetone, and petroleum ether) were purchased from VWR (Milan, Italy). 

Hydrogenated Sunflower Oil (HSO) (VGB 5 ST, free fatty acids 0.07%) was a gift from ADM-SIO (Saint-Laurent-Blangy, France), an ADM company. β-carotene, stored under argon, was purchased from TCI (Tokyo Chemical Industry Co., Ltd.) (Toshima, Kitaku, Tokyo, Japan) (purity >97.0%; m. p. 184 °C). Soy lecithin, monobasic sodium phosphate, and dibasic sodium phosphate were purchased from VWR (Milan, Italy), and cholic acid sodium salt (99% purity) was purchased from Acros Organics (Geel, Belgium). Ultrapure water was generated by Synergy^®^ UV Water Purification System (Millipore Sigma, St. Louis, MI, USA).

### 3.3. Extraction Methods of Carotenoids from Pumpkin Pulp 

Before each extraction (UAE, MAE, MAC), the dried sample was subjected to a static soaking process for 15 min at room temperature in order to guarantee the solvent penetrated the vegetable matrix. After this time, the sample is gently vortexed for about 5 s before being subjected to the extraction processes.

#### 3.3.1. Ultrasound-Assisted Extraction (UAE)

The extraction procedure was carried out following the conditions of previous papers [16,17] with slight modifications. A dried pumpkin sample (1 g) was extracted with 20 mL of solvent (hexane:isopropanol, 60:40 *v/v* or hexane: acetone: ethanol 50:25:25 *v/v/v*) for 30 min at 45 °C using a sonication bath (mod. AU-65, ArgoLab, Carpi, Italy). The temperature was carefully monitored every 10 min, adding ice when 45 °C was exceeded. The ultrasonic power was 180 W. The extracts were then filtered through a paper filter (MN 615, Macherey–Nagel, Düren, Germany), collected in amber glass vials, and kept at −20 °C until further analysis. The extraction was repeated three times.

#### 3.3.2. Microwave-Assisted Extraction (MAE)

The extraction procedure was carried out following the conditions of previous papers [16,17] with slight modifications. A dried pumpkin sample (1 g) was extracted with 20 mL of solvent (hexane:isopropanol, 60:40 *v/v* or hexane: acetone: ethanol, 50:25:25 *v/v/v*) for 15 min or 30 min at 45 °C using a closed vessel system microwave (Model Initiator 2.0, version 2.3, Biotage AB, Uppsala, Sweden) under controlled conditions. The temperature was the preferred controlled variable to avoid degradation of the target compounds and to achieve maximum efficiency. The other parameters were directly dependent on the temperature, such as the magnetron power (maximum 40 W) and pressure (maximum 5 bar). At the end of the treatment, the vessel used was cooled to room temperature. The extracts were filtered through a paper filter (MN 615, Macherey–Nagel, Düren, Germany), collected in amber glass vials, and kept at −20 °C until further analysis. The extraction was repeated three times.

#### 3.3.3. Maceration (MAC)

The extraction procedure was carried out following the conditions of previous papers [16,17] with slight modifications. A dried pumpkin sample (1 g) was extracted for 4 h min at room temperature while being stirred, using a dynamic maceration with 20 mL of solvent (hexane:isopropanol, 60:40 *v/v* or hexane: acetone: ethanol, 50:25:25 *v/v/v*). The extracts were filtered through a paper filter (MN 615, Macherey–Nagel, Düren, Germany), collected in amber glass vials, and kept at − 20 °C until further analysis. The extraction was repeated three times.

### 3.4. Determination of Total Carotenoids Content (TCC)

The total carotenoid content of carotenoid extracts from pumpkins was measured by the method suggested by Sharma and Bhat [17] with minor modifications. An aliquot of the sample was taken, the solvent completely removed, and the sample suspended in a known volume of petroleum ether such as to obtain an absorbance reading of less than 1. The quantification was made using a calibration curve (y = 200.31x + 0.0026; R² = 0.9993) constructed by using the β-carotene standard in the concentration range of 0.001–0.005 mg/mL. A known quantity of standard was re-weighed in a known volume of Petroleum Ether, and subsequent dilutions were used to construct the calibration curve. The solutions were assessed for absorbance value (A) by using a spectrophotometer (Lambda 20 spectrophotometer, PerkinElmer, Inc; Waltham, MA, USA) at 450 nm. 

### 3.5. In Vitro Antioxidant Activities

#### 3.5.1. Free Radical-Scavenging Activity Using DPPH (DPPH Assay)

DPPH assay was carried out according to the procedure described by a previous papers [48]. DPPH methanolic solution was added to each extract. The change in the absorbance of the sample extract was measured at 515 nm after 30 min. The antioxidant capacity of each sample was expressed as μg Trolox equivalents (TE) per gram of dry pumpkin pulp (μg TE/g).

#### 3.5.2. Free Radical-Scavenging Activity Using ABTS (ABTS Assay)

As regards the evaluation of the antioxidant properties of extracts, the ABTS assay was performed according to the procedure described by a previous paper [49]. A freshly prepared ABTS+ solution was added to the sample extracts, and the absorbance was measured at 734 nm after 10 min. The antioxidant capacity of each sample was expressed as μg TE/g of dry pumpkin pulp.

As regards the evaluation of the antioxidant properties of β-carotene encapsulated in SLN, the assay was performed in accordance with the procedure of Durmaz [46]. The reagent was freeze-dried overnight, and then the obtained radical cation powder was dissolved and diluted in chloroform:methanol (1:1 *v/v*) to an absorbance of 0.700 (± 0.030). The absorbance was determined at 752 nm by UV spectrophotometer using the chloroform-methanol mixture as a blank. Taking into account its antioxidant property, samples containing only soy lecithin at the same concentration as in the SLN suspension were tested to ensure that the recorded activity was related to the sole β-carotene. The antioxidant capacity of the sample was expressed as mg of Trolox equivalents/mg SLN and determined from a calibration curve prepared with Trolox solutions in chloroform:methanol (1:1 *v/v*) (0.3–50 μg/mL) previously treated using the same procedure as for the SLN suspension. For each standard solution, the absorbance was measured in triplicate at 752 nm after 10 min of incubation with the ABTS^•+^. The antioxidant activity was expressed in terms of mg of Trolox equivalents/mg SLN. SLN loaded with 0.5 and 1% β-carotene were analyzed. In particular, three different batches of SLN (loaded with 0.5 or 1% β-carotene) prepared on three different days were analyzed in duplicate. As for the Trolox standard solutions, the absorbance was read in triplicate for each sample.

#### 3.5.3. FRAP

FRAP assay was carried out according to the procedure described by Rocchetti et al. [16]. The FRAP reagent was added to the sample extract, and the reaction mixture was kept in the dark for 4 min at room temperature. The corresponding absorbance was measured at 593 nm. The antioxidant capacity of each sample was expressed as μg TE/g of dry pumpkin pulp.

#### 3.5.4. Oxygen Radical Absorbance Capacity (ORAC) Assay (ORAC Assay)

The ORAC assay, carried out to assess the TAC of the extracts, was performed according to the procedure described by Rocchetti et al. [16]. The method is based on the production of peroxyl free radicals generated by the thermal decomposition of an azo-compound, AAPH, with the fluorescence of a fluorescein probe. These radicals quench the fluorescence of the probe, and the level of reduction depends on the antioxidant that acts as a quencher of the produced radicals. The radiative decay of the fluorescein fluorescence was determined by a high-performance plate reader (FLUOstar Optima, BMG Labtech, Germany). The areas under the curves (AUCs) of fluorescence decay of Trolox were selected as the blank (reference standard), and extracts were used to quantify the TAC (expressed as µmol TE/g). The readings of fluorescence intensity were carried out on the spectrofluorometer at excitation and emission wavelengths of 485 and 520 nm, respectively. The net AUC was calculated by subtracting the blank AUC from the AUC of each extract, the standards, and the positive control. Final ORAC values, reported as the equivalent concentration of Trolox that produces the same level of antioxidant activity as the samples at 20 mg/mL, were calculated as follows:

ORAC = [C_Trolox_ × (AUC_sample_ − AUC_blank_) × k]/(AUC_Trolox_ − AUC_blank_) (2)

where C_Trolox_ is the Trolox concentration, k is the sample dilution factor, and AUC is the area under the fluorescence decay curve.

### 3.6. HPLC-DAD Analysis of Carotenoids and Method Validation 

The HPLC measurements were made on a Thermo Separation low-pressure quaternary gradient pump system coupled to a Spectra system UV 6000 LP diode array detector (DAD) (Thermo Scientific, Waltham, MA, USA), supplied with a GT-154 vacuum degasser (Shimadzu, Kyoto, Japan), and a Rheodyne7725i injector (Rheodyne Inc., Cotati, CA, USA) with a 20 μL stainless steel loop. Excalibur software (Chromatographic Specialties Inc., Canada) was used for data acquisition.

Chromatographic separation was performed on a reverse-phase C30 Develosil column (250 × 4.6 mm i.d., 5 μm particle size, Nomura Co., Kyoto, Japan). 

The optimized gradient program was obtained from eluent A (methanol:water, 97:3 *v/v*) and eluent B (MTBE) as follows: 0 min 84% A, 11.25 min up to 78% A, 30 min up to 55% A, 60 min up to 65% A. The employed mobile phase components were degassed by sonication for 15 min before use and flowed through the column at a 1.0 mL/min flow rate. A column re-equilibration step for 20 min was added between consecutive runs. The detection of carotenoids was followed at 450 nm. Analyses were performed in triplicate.

Quantitation of carotenoids (α- and β-carotene) in the extracts was performed by relying upon a calibration curve properly constructed by plotting β-carotene standard concentrations against the measured response (peak area value). In total, 5 calibration solutions were prepared with concentration values spanning in the range of 0.51–51 µg/mL. 

The optimized HPLC method was validated in terms of precision and accuracy in both the short- (intra-day) and the long-term (inter-day) period, the limit of detection (LOD) and the limit of quantification (LOQ). 

A β-carotene control solution (with a theoretical concentration fixed at 4.1 μg/mL) was run in triplicate on the same day and for three different days. The method precision was determined as the relative standard deviation (RSD%) among the concentration values obtained from consecutive injections within the short- or long-term period. The same procedure was followed for the evaluation of the method accuracy, expressed as a percentage of the β-carotene recovery as follows:

Recovery% = (C_measured_/C_theoretical_) × 100 (3)

The LOD and LOQ concentrations (C_LOD_ and C_LOQ_) were calculated according to the following Equations (4) and (5):

C_LOD_ = 3.3 × (σ_y_/b) (4)

C_LOQ_ = 10 × (σ_y_/b) (5)

where σ_y_ is the standard deviation of the response and b is the slope of the calibration curve.

### 3.7. LC-HRMS Analysis for Carotenoids Structural Confirmation

LC-MS/MS analyses for the structural confirmation of isolated peaks of both α- and β-carotene were made on an Agilent 1290 Infinity LC system coupled with an Agilent 6560 Ion Mobility Q-TOF LC/MS (Agilent Technologies, Santa Clara, CA, USA) equipped with a Dual Agilent Jet Stream Electrospray Ionization (Dual AJS ESI) source and controlled by the Mass Hunter Workstation software 4.0 (MH) from Agilent. 

The mass spectrometer was operated in MS and MS/MS modes setting the following parameters: source gas temperature of 275 °C, drying gas flow of 12 L/min, nebulizer pressure of 35 psi, sheath gas temperature of 325 °C, and sheath gas flow of 12 L/min. VCap voltage was set at 3500 V, nozzle voltage 250 V, fragmentor voltage 90 V, octopole RF peak at 750 V, and MS/MS collision energy of 20 V. The instrument was operated in positive ion mode; full scan spectra were obtained in the range of *m*/*z* 100–1000. The reference compound solution for internal mass calibration of the Q/TOF at mass spectrometer contained 5 μM of purine (121.0509 *m/z*) and 2.5 μM HP-0921 (922.0098 *m/z*) in acetonitrile:water (95:5 *v/v*) from Agilent.

The chromatographic method employed a Zorbax Eclipse Plus C18 column (2.1 × 50 mm, 1.8 μm from Agilent Technologies, Santa Clara, CA, USA) maintained at 30 °C. The flow rate was 0.5 mL/min; the injection volume was 5 μL. The gradient program was obtained from eluent A (water with 0.1% formic acid) and eluent B (methanol:acetonitrile:isopropanol, 2:2:1 *v/v/v*) with 0.1% formic acid as follows: 0 min 80% B, 6 min up to 97% B, 16 min 97% B, 17 min return to initial conditions with 80% B and re-equilibration for 5 min. Isolated samples, and β-carotene commercial standard, were resuspended in methanol, and a sample volume of 5 μL was injected. 

### 3.8. Solid Lipid Nanoparticles Preparation

Hot, high-pressure homogenization (HPH) was employed to prepare solid lipid nanoparticles (SLN). Briefly, HSO (400 mg) was melted in a water bath at ~75 °C, and then, 0.8% *w*/*v* soy lecithin was added to the lipid phase under magnetic stirring until a good homogeneity was reached. A buffer solution (pH = 7) was prepared by dissolving 4 mM monobasic sodium phosphate and 4 mM dibasic sodium phosphate in ultrapure water. Then, the molten lipid phase was added to the heated (~75 °C) aqueous buffer (20 mL) containing sodium cholate (0.3% *w*/*v*) by utilizing a heated Pasteur pipette. The pre-emulsion was obtained by processing the mixture with an Ultraturrax homogenizer (model T25 basic, IKA^®^-Werke GmbH & Co. KG, Staufen, Germany) at 8000 rpm for 1 min, keeping the coarse emulsion in a hot bath. Immediately after, the pre-emulsion was subjected to 5 homogenization cycles at 1000 bar using an Avestin EmulsiFlex-C5 High-Pressure Homogenizer, thermostated at 75 °C. After the first two cycles, the feed cylinder was cleaned to remove the remaining larger particles. The colloidal emulsion was finally cooled in an ice bath to solidify the lipid, and a suspension of SLN in the aqueous buffer was obtained. 

Loaded SLN were produced by adding different amounts of β-carotene, i.e., 0.5, 1, 5, and 10% (*w*/*w*), in the lipid phase. the β-carotene powder was added to the molten HSO under magnetic stirring until obtaining a homogeneous phase. Thereafter, the procedure used to produce blank SLN was followed.

### 3.9. Solid Lipid Nanoparticles Characterization

#### 3.9.1. Particle Size Determination

Samples were analyzed using Dynamic Light Scattering (DLS) instrument (Zeta potential/particle sizer NICOMP 380 ZLS (Santa Barbara, CA, USA)). In particular, 50 µL of suspension was transferred to a cuvette that was then filled up with about 3 mL of fresh ultrapure water. Samples were left to stabilize for 10 min before running the analysis to reach the temperature set by the instrument. Refractive index of 1.33 for the dispersant and a laser at 658 nm wavelength were used. All measurements were carried out at 23 °C, and data were collected for 12 min. Data were expressed as mean diameter ± standard deviation (SD) using the NICOMP, based on the variation in the intensity of scattered light (INTENSITY-WT).

#### 3.9.2. β-Carotene Quantification

Starting with a stock solution of pure β-carotene in n-hexane, sequential dilutions were made in duplicate, and the absorbance at 450 nm was read in triplicate using an Agilent 8453 UV-Vis Spectrophotometer (Agilent Technologies, Waldbronn, Germany). The calibration curve was linear, with a correlation coefficient R^2^ of 0.99227, over a concentration range of 0.5–5.0 µg/mL. The overall amount of β-carotene loaded in the suspension was determined by drying 200 µL of SLN and solubilizing the residue in a known volume of n-hexane (10 mL). Then, solutions were analyzed using a UV-Vis spectrophotometer to determine the β-carotene concentration. Finally, the encapsulation efficiency (*EE*) of SLN was determined using the following equation:

(6) $$\%EE=\frac{\beta -\mathrm{carotene}\;\mathrm{quantified}\;\mathrm{in}\;\mathrm{the}\;\mathrm{SLN}}{\beta -\mathrm{carotene}\;\mathrm{initially}\;\mathrm{added}\;\mathrm{to}\;\mathrm{the}\;\mathrm{lipid}}\times \;100$$

#### 3.9.3. Morphological Analysis

SLN morphology was characterized using transmission electron microscopy (TEM) (PhilipsEM400Tmicroscope, Eindhoven, Netherlands). Samples were prepared by allowing a drop of the SLN suspension to dry overnight at 25 °C, keeping it covered to avoid dust deposition on the surface of a 200 mesh Formvar^®^ coated copper grid (TAAB Laboratories Equipment Ltd., Aldermaston, UK). Dried SLN were stained using one drop of phosphotungstic acid (PTA) solution (1%) and dried overnight at 25 °C, keeping it covered to avoid dust deposition prior to TEM investigation.

### 3.10. Statistical Analysis

All analytical determinations (yield, TCC, antioxidant assay, chromatographic data) were performed in triplicate, and the results, expressed as the mean ± standard deviation (SD), were reported on dried weight (DW). Microsoft Excel 2016 (Microsoft Corporation, Redmond, WA, USA) was used for data analysis. A *p*-value ≤ 0.05 was considered to be significant.

## 4. Conclusions

In this research, the impact of different extraction technologies (UAE, MAE, and MAC) in terms of carotenoid recovery from pumpkin pulp by using ternary or binary solvent mixtures was evaluated. The results of this study reveal the unconventional UAE as an efficient extraction technique belonging to the so-called green chemistry. UAE allowed the extraction of non-polar carotenoids, in particular β-carotene and α-carotene requiring a low amount of solvent and energy cost and preserving their integrity. The results obtained allow us to state that the extraction is a crucial step in the analytical procedure and show that pumpkin pulp represents an interesting source of carotenoids, with potential use for the design of functional foods or nutraceuticals. Taking into consideration all analytical parameters and their correlations, it can be confirmed that the unconventional UAE with the binary mixture was the most successfully extracting technique for the isolation of total non-polar carotenoids from pumpkin pulp, while the unconventional MAE cannot be recommended for this aim.

β-carotene was successfully encapsulated in SLN stabilized by soy lecithin and sodium cholate. The SLN obtained had suitable dimensions of a few hundred nanometers when β-carotene (0.5 and 1% *w*/*w*) was added to the lipid phase. The preservation of the antioxidant activity of β-carotene exposed to the technological processes was also demonstrated. The extraction of the oil from pumpkin seeds, another interesting pumpkin waste, is already in progress. It will be used, together with HSO, to prepare SLN in order to enhance the loading capacity of β-carotene and extract. The physical stability (i.e., dimensions and loading) of SLN will also be investigated. To favor SLN handling and stability, the optimization of freeze-drying conditions will be carried out.

## Figures and Tables

**Table 1:** Yield (%) of extraction, TCC and in vitro antioxidant activities (ABTS and ORAC assays) of extracts.

Extraction Technique	Extraction Solvent	Yield (%)	TCC (μg/g)	ABTS (μg TE/g)	ORAC (μg TE/g)
UAE	Hex:Iso 60:40	2.30 ± 0.00	145.33 ± 2.04	958.88 ± 24.90	2832.76 ± 24.56
	Hex:Ac:EtOH 50:25:25	2.45 ± 0.01	138.14 ± 6.95	2332.32 ± 11.99	2512.11 ± 35.12
MAE	Hex:Iso 60:40	2.73 ± 0.02	126.02 ± 3.97	505.01 ± 26.51	1955.88 ± 15.20
	Hex:Ac:EtOH 50:25:25	3.13 ± 0.03	118.76 ± 9.49	1870.03 ± 22.79	1732.28 ± 20.71
MAC	Hex:Iso 60:40	2.63 ± 0.01	135.64 ± 8.76	801.25 ± 34.39	2586.12 ± 21.50
	Hex:Ac:EtOH 50:25:25	3.08 ± 0.00	162.81 ± 3.42	3697.62 ± 29.51	2495.12 ± 33.27

Data are reported as mean ± standard deviation (SD) of three independent measurements (n = 3) and are expressed on dry weight (DW). UAE, ultrasound-assisted extraction; MAE, microwave-assisted extraction; MAC, maceration; TCC, total carotenoid content measured by spectrophotometric assay; ABTS, 2,2′-azino-bis(3-ethylbenzothiazoline-6-sulfonic acid) diammonium salt; ORAC, Oxygen Radical Absorbance Capacity; Hex:Iso, Hexane:Isopropanol, 60:40 *v/v*; Hex:Ac:EtOH, Hexane:Acetone:Ethanol, 50:25:25 *v/v/v*.

**Table 2:** Regression equation, R^2^, limit of detection (LOD), and limit of quantification (LOQ) of β-carotene analyzed by HPLC-DAD.

Regression Equation	R^2^	Linearity Range (μg/mL)	LOD (ng/mL)	LOQ (ng/mL)
y = 179307 (±2636.77)x + 144.96 (±58.18)	0.999	0.51–51.00	1.07	3.24

**Table 3:** Method validation for β-carotene: evaluation of precision (RSD %) and accuracy (Recovery %) in the short- and long-term period (intra-day and inter-day precision and accuracy values).

Theoretical Conc. (µg/mL)	Intra-Day Mean Conc. (µg/mL)	Intra-Day Precision (RSD%)	Intra-Day Accuracy (Recovery%)	Inter-Day Mean Conc. (µg/mL)	Inter-Day Precision (RSD%)	Inter-Day Accuracy (Recovery%)
4.1	4.20	2.12	102.34	4.28	0.11	104.46
	4.33	3.42	105.68			
	4.32	0.60	105.36			

Intra-day and Inter-day evaluation: analysis of 3 replicates of the selected external standard within one day and for three consecutive days.

**Table 4:** Content (μg/g, DW) of α-carotene, β-carotene, and total non-polar carotenoids in the extracts.

Extraction Technique	Extraction Solvent	α-Carotene μg/g	β-Carotene μg/g	α- and β-Carotene μg/g
UAE	Hex:Iso	684.23 ± 55.36	893.00 ± 70.18	1577.24 ± 131.12
	Hex:Ac:EtOH	766.03 ± 22.26	987.11 ± 11.34	1753.14 ± 128.45
MAE	Hex:Iso	509.16 ± 35.18	662.93 ± 36.73	1172.08 ± 93.51
	Hex:Ac:EtOH	637.31 ± 9.17	831.60 ± 18.09	1468.91 ± 112.79
MAC	Hex:Iso	633.33 ± 10.46	816.82 ± 17.68	1450.15 ± 106.60
	Hex:Ac:EtOH	795.95 ± 75.00	1038.34 ± 72.83	1834.29 ± 152.41

Data are reported as mean value ± standard deviation (SD) of three independent measurements (n = 3) and are expressed on dry weight (DW).

**Table 5:** Correlation between all analytical parameters (TCC, ABTS, ORAC, and HPLC-DAD) used to characterize all the extracts (UAE, MAE, and MAC), taking into consideration binary or ternary mixtures.

		TCC	ABTS	ORAC	HPLC-DAD
TCC	Hex:Iso	-			
	Hex:Ac:EtOH	-			
ABTS	Hex:Iso	0.9692	-		
	Hex:Ac:EtOH	0.9571	-		
ORAC	Hex:Iso	0.8844	0.9712	-	
	Hex:Ac:EtOH	0.6700	0.4649	-	
HPLC-DAD	Hex:Iso	0.9550	0.9986	0.9824	-
	Hex:Ac:EtOH	0.8625	0.6919	0.9471	-

**Table 6:** Mean particle size of SLN loaded with different percentages of β-carotene.

β-Carotene (%, *w*/*w*)	Mean Diameter ^x^ ± SD (NICOMP, nm)
0.5	93.4 ± 17.2 (11.0%)
	316.7 ± 70.4 (89.0%)
1	98.7 ± 14.4 (13.6%)
	363.7 ± 65.8 (86.4%)
5	99.1 ± 15.0 (10.3%)
	447.2 ± 90.0 (89.7%)
10	147.9 ± 28.2 (14.2%)
	711.1 ± 144.4 (85.8%)

^x^ Particle sizes were determined using dynamic light scattering (DLS) NICOMP 380 ZLS apparatus. The dimensional data are expressed using the NICOMP, based on the variation in the intensity of scattered light (INTENSITY-WT). The relative percentage of the different populations is indicated between brackets.

**Table 7:** β-carotene content and encapsulation efficiency of loaded SLN.

β-Carotene Theoretical Content (%, *w*/*w*)	β-Carotene Experimentally Determined Content ^y^ ± SD (%, *w*/*w*)	EE ^y^ (%)
0.54 ± 0.01	0.49 ± 0.02	89.57
1.01 ± 0.01	0.75 ± 0.09	74.56

^y^ Experimentally determined content and encapsulation efficiency were determined on three different batches of SLN prepared on different days.

**Table 8:** Antioxidant capacity measured with the ABTS assay of the SLN formulations.

Sample	ABTS Assay ^z^ (mg Trolox eq./mg SLN)	
	Average ± SD	RSD%
SLN 0.5%	0.050 ± 0.005	9.88
SLN 1%	0.061 ± 0.006	10.05

^z^ Data were obtained on three different batches of SLN prepared on different days.

## Data Availability

Not applicable.

## Supplementary Materials

The following supporting information can be downloaded at: https://www.mdpi.com/article/10.3390/molecules27238240/s1, Figure S1: Fragment ion spectrum for β-carotene commercial standard; Figure S2: Fragment ion spectrum for β-carotene isolated by HPLC; Figure S3: Fragment ion spectrum for α-carotene isolated by HPLC.

## Sample Availability

Samples of the compounds are not available from the authors.
