# Supplementary material for: Unconventional Extraction of Total Non-Polar Carotenoids from Pumpkin Pulp and Their Nanoencapsulation
Source: Molecules. 2022 Nov 25;27(23):8240. doi: 10.3390/molecules27238240 (PMC9736262; doi:10.3390/molecules27238240)
Supplement: Supplementary file 1 [file molecules-27-08240-s001.zip › molecules-1985429-supplementary.pdf]

molecules

Supplementary Material

Unconventional Extraction of Total Non-Polar Carotenoids from Pumpkin Pulp and their Nanoencapsulation

Nicola Pinna [1,†], Federica Ianni [1,†], Francesca Blasi [1,*], Arianna Stefani [2], Michela Codini [1], Stefano Sabatini [2], Aurélie Schoubben [2,*] and Lina Cossignani [1,3]

[1] Department of Pharmaceutical Sciences, Section of Food Sciences and Nutrition, University of Perugia, 06126 Perugia, Italy

[2] Department of Pharmaceutical Sciences, Section of Pharmaceutical Chemistry and Technology, University of Perugia, 06123 Perugia, Italy

[3] Center for Perinatal and Reproductive Medicine, Santa Maria della Misericordia University Hospital, University of Perugia, 06132 Perugia, Italy

* Correspondence: francesca.blasi@unipg.it (F.B.); aurelie.schoubben@unipg.it (A.S.); +39-075-585-7954 (F.B.); +39-075-585-2057 (A.S.)

† These authors have equally contributed

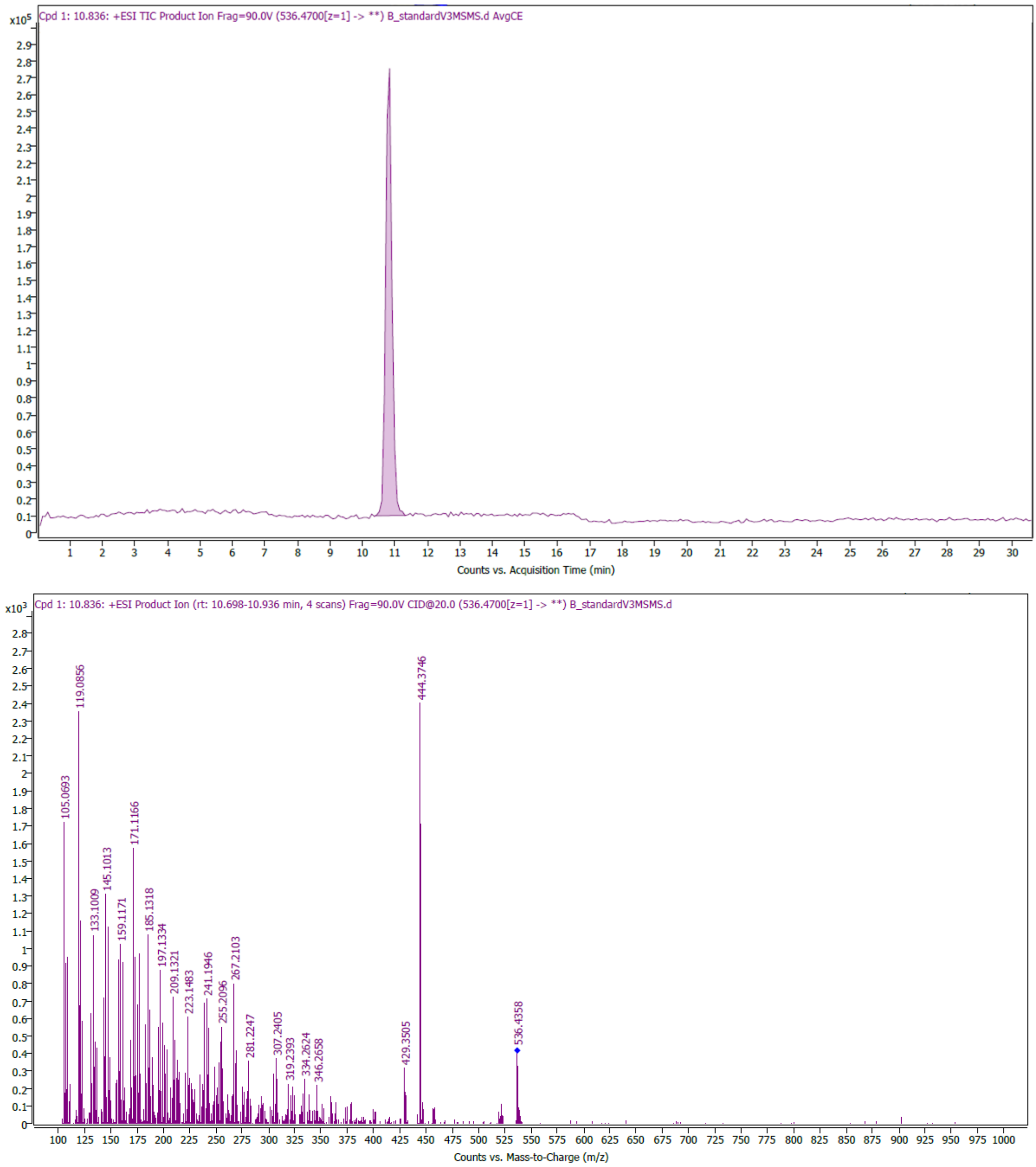

Figure S1. Fragment ion spectrum for β-carotene commercial standard.

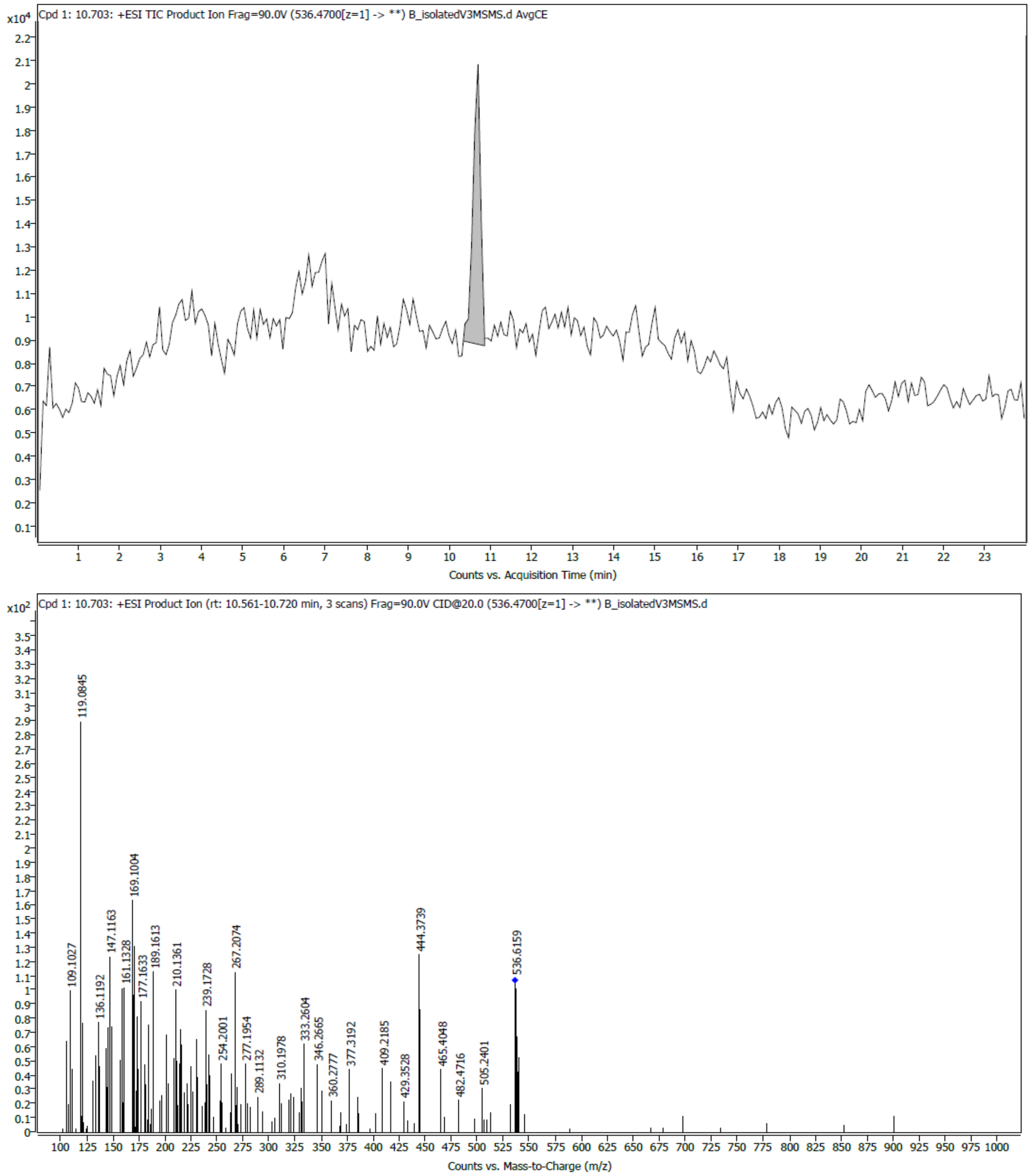

Figure S2. Fragment ion spectrum for β-carotene isolated by HPLC.

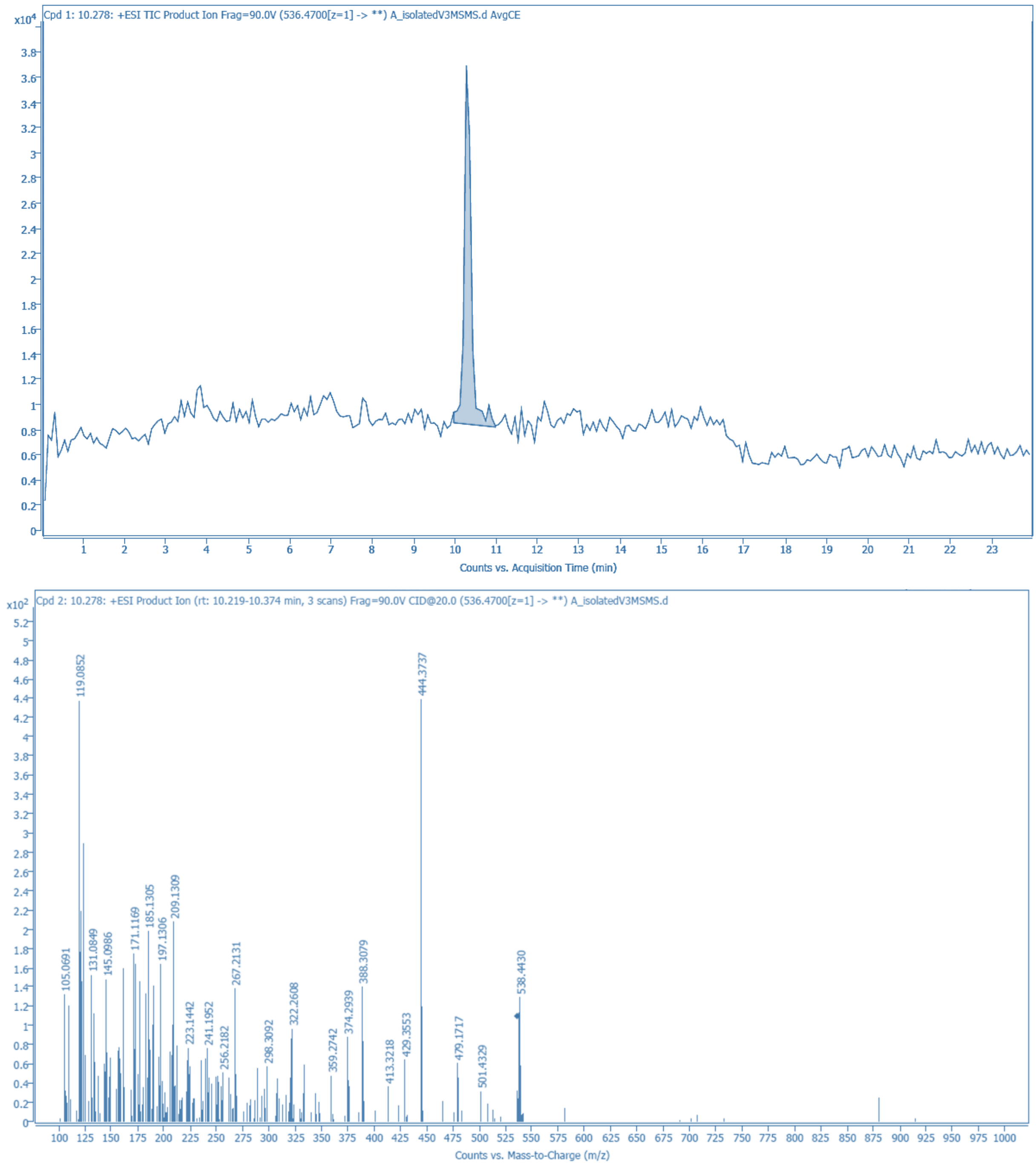

Figure S3. Fragment ion spectrum for α-carotene isolated by HPLC.
